# Mid-treatment MRI-based tumor volume reduction rate as a continuous prognostic factor after chemoradiation for cervical cancer: development and two-center internal–external validation

**DOI:** 10.1093/jrr/rrag043

**Published:** 2026-06-25

**Authors:** Takashi Saito, Keiritsu Sho, Tsukasa Saida, Taisuke Sumiya, Keiichiro Baba, Motohiro Murakami, Haruko Numajiri, Ayumi Shikama, Masashi Mizumoto, Kazushi Maruo, Akinori Oki, Toshiyuki Okumura, Yoshio Tamaki, Toyomi Satoh, Hideyuki Sakurai

**Affiliations:** Department of Radiation Oncology, Institute of Medicine, University of Tsukuba, Tsukuba, Ibaraki 305-8575, Japan; Department of Radiation Oncology, Institute of Medicine, University of Tsukuba, Tsukuba, Ibaraki 305-8575, Japan; Department of Diagnostic and Interventional Radiology, Institute of Medicine, University of Tsukuba, Tsukuba, Ibaraki 305-8575, Japan; Department of Radiation Oncology, Institute of Medicine, University of Tsukuba, Tsukuba, Ibaraki 305-8575, Japan; Department of Radiation Oncology, Institute of Medicine, University of Tsukuba, Tsukuba, Ibaraki 305-8575, Japan; Department of Radiation Oncology, Ibaraki Prefectural Central Hospital, Kasama, Ibaraki 309-1793, Japan; Department of Radiation Oncology, Institute of Medicine, University of Tsukuba, Tsukuba, Ibaraki 305-8575, Japan; Department of Obstetrics and Gynecology, Institute of Medicine, University of Tsukuba, Tsukuba, Ibaraki 305-8575, Japan; Department of Radiation Oncology, Institute of Medicine, University of Tsukuba, Tsukuba, Ibaraki 305-8575, Japan; Department of Biostatistics, Institute of Medicine, University of Tsukuba, Tsukuba, Ibaraki 305-8575, Japan; Department of Obstetrics and Gynecology, Ibaraki Prefectural Central Hospital, Kasama, Ibaraki 309-1793, Japan; Department of Radiation Oncology, Ibaraki Prefectural Central Hospital, Kasama, Ibaraki 309-1793, Japan; Department of Radiation Oncology, Ibaraki Prefectural Central Hospital, Kasama, Ibaraki 309-1793, Japan; Department of Radiation Oncology, Fukushima Rosai Hospital, Iwaki, Fukushima 973-8403, Japan; Department of Obstetrics and Gynecology, Institute of Medicine, University of Tsukuba, Tsukuba, Ibaraki 305-8575, Japan; Department of Radiation Oncology, Institute of Medicine, University of Tsukuba, Tsukuba, Ibaraki 305-8575, Japan

**Keywords:** uterine cervical neoplasms, chemoradiotherapy, magnetic resonance imaging, prognostic models, tumor volume reduction rate, brachytherapy

## Abstract

This study aimed to quantify the continuous association of the mid-treatment MRI-based tumor volume reduction rate (TVRR) with oncologic outcomes after chemoradiation for cervical cancer and to develop prognostic models assessed by two-center internal-external cross-validation (IECV). A retrospective analysis was performed in patients with FIGO 2009 stage IB–IVA disease treated with definitive chemoradiation at two centers from 2010 to 2021 with both pre- and mid-treatment pelvic MRI during external beam radiotherapy (24–36 Gy). Pre- and mid-treatment volumes (*V*_pre_, *V*_mid_) were derived and TVRR was defined as (*V*_pre_ − *V*_mid_)/*V*_pre_ × 100%. Cox models were used to evaluate overall survival (OS) and progression-free survival (PFS), and a Fine-Gray model was used for local control (LC), modeling TVRR with a penalized spline and adjusting for key covariates. IECV assessed discrimination and 60-month calibration for OS and PFS. The analysis included 281 patients (median age 59 years; squamous cell carcinoma 85%; FIGO 2009 stage III–IV 60%; node-positive 56%) with 65 deaths, 97 PFS events, and 27 local failures over a median follow-up period of 69.6 months. Higher TVRR was independently associated with better outcomes: per 10-percentage-point (pp) increase, OS hazard ratio (HR) 0.71 (95% CI, 0.64–0.79; *P* < 0.001), PFS HR 0.74 (0.68–0.81; *P* < 0.001), and LC subdistribution HR 0.68 (0.58–0.80; *P* < 0.001). Non-linear components were not significant. Discrimination was moderate-high (C 0.73–0.81 internally; 0.72–0.79 in IECV) with acceptable 60-month calibration. In conclusion, mid-treatment MRI-based TVRR provides a strong, near-linear prognostic signal and enables threshold-free, calibrated risk estimation and supports design of risk-adapted trials.

## INTRODUCTION

The tumor response to radiotherapy (RT) has long been prognostic in cervical cancer. MRI performed during or after external-beam radiotherapy (EBRT) consistently shows that greater tumor shrinkage and smaller residual volume are associated with better overall survival and local control, often revealing significant divergence in outcomes [1, 2]. This likely reflects underlying tumor biology: faster volumetric regression marks a more radio-responsive tumor, whereas a larger residual burden indicates relative radio-resistance [2].

The principal limitation is threshold-based categorization of a continuous measure such as tumor volume reduction rate (TVRR). Clinically, this turns a smooth risk gradient into a cliff: two otherwise similar patients with, for example, 59% vs. 61% shrinkage, are pushed to opposite risk labels even though their true prognoses should be almost the same. This also leads to loss of information, since a median split retains only about two-thirds of the prognostic signal, making models less sensitive [3]. Other practical challenges include how shrinkage is measured and how interobserver variability is handled. Multicenter delineation exercises have shown that derived planning targets have substantial between-observer variability even after training on a unified guideline, showing the need for standardization of imaging planes and region of interest (ROI) rules [4].

To address these limitations, this study was designed to quantify the continuous association between mid-treatment MRI-based TVRR and oncologic outcomes in cervical cancer treated with definitive chemoradiation. By prespecifying spline-based time-to-event models and implementing two-observer three-dimensional (3D) volumetry, we robustly characterize the continuous prognostic gradient of TVRR, providing clinically interpretable effect estimates that inform risk-stratified, adaptive care.

## METHODS

### Study design and ethics

A retrospective, two-center cohort study was conducted at Centers A and B in a single healthcare region. A generalizability summary of the study population, setting, measurement and treatment is provided in Supplementary Table S1. The protocol was approved by the ethics committee of Tsukuba Clinical Research & Development Organization (R05-088) and the Institutional Review Board of Ibaraki Prefectural Central Hospital (1699) and the study was conducted in accordance with the Declaration of Helsinki. Written consent for clinical care was obtained from all patients. The requirement for written informed consent for this retrospective analysis was waived via an IRB-approved opt-out process. All data were de-identified prior to analysis.

### Institutional setting

Center A was University of Tsukuba Hospital and Center B was Ibaraki Prefectural Central Hospital. High-dose-rate brachytherapy (BT) was available at both centers with interstitial capability, including hybrid intracavitary-interstitial (IC/IS) and interstitial brachytherapy (ISBT) techniques. In this healthcare region, more complex referrals tended to be directed to Center A. In some cases, the need for advanced IC/IS or ISBT was first recognized at mid-treatment MRI, and these patients were subsequently referred to Center A for BT while EBRT continued at the original institution. EBRT prescription and fractionation were standardized according to the protocol of the BT center.

### Patient selection

Consecutive patients were identified who were treated at each center after implementation of mid-treatment MRI (Center A, 2014–21; Center B, 2010–21) and had pathologically confirmed cervical cancer and received definitive chemoradiation. Patients treated with RT alone were excluded because TVRR reflects tumor biological response under a given treatment, and mixing treatment backbones would confound the prognostic interpretation of TVRR. Eligibility was defined as International Federation of Gynecology and Obstetrics (FIGO) 2009 stage IB–IVA without distant metastasis at baseline. Exclusion criteria included induction chemotherapy prior to RT; concomitant immune-checkpoint inhibitor (ICI) therapy; and absence of, or inadequate-quality, pelvic MRI at the pre- or mid-treatment time point.

### Imaging acquisition and adequacy

Image-quality selection criteria were defined a priori based on European Society of Urogenital Radiology (ESUR) MRI guidelines [5, 6]. Volumetry used a prespecified T2-weighted working series (axial-oblique preferred; axial/sagittal acceptable) fixed across time points with an orthogonal T2 for cross-reference. Pre-treatment MRI was generally obtained within 4 weeks before the start of RT. Mid-treatment MRI was performed during EBRT at ~30 Gy (permitted window, 24–36 Gy). Detailed parameters (slice thickness/gap, artifact criteria) are provided in Supplementary Method S1.

### ROI delineation and tumor volumetry

Before segmentation, the ESUR MRI guidelines were consolidated into a study-specific protocol specifying T2-based anatomical boundaries and plane-selection rules (details in Supplementary Method S2) [5, 6]. Blinded to oncologic outcomes, two radiation oncologists (observer A, 13 years’ experience in gynecologic oncology; observer B, 3 years’ experience) delineated the MRI-based gross tumor volume (GTV), which was defined as the macroscopic primary cervical tumor. A diagnostic radiologist with 22 years’ experience and subspecialty training in gynecologic imaging participated in the consensus review (Section 2.6). Pre-treatment tumor volume (*V*_pre_) was defined as the GTV volume on pre-treatment MRI, and mid-treatment tumor volume (*V*_mid_) as the GTV volume on mid-treatment MRI. Volumes were calculated in RayStation (RaySearch Laboratories AB, Stockholm, Sweden) software by counting voxels within the contoured 3D ROI and multiplying by voxel size. TVRR was calculated as (*V*_pre_ − *V*_mid_)/*V*_pre_ × 100%.

### Consensus review and interobserver agreement

After independent segmentations, cases were triaged to consensus review by two prespecified triggers: (i) a subjective flag and (ii) any inter-observer difference in *V*_pre_, *V*_mid_ or TVRR falling outside nonparametric limits of agreement calculated from the study data according to a prespecified rule. Consensus review was conducted jointly by the observers and diagnostic radiologist, followed by recontouring as agreed. Definitive *V*_pre_ and *V*_mid_ were taken as the mean of the two observers’ contours. Final TVRR was recomputed from these definitive volumes. An intraclass correlation coefficient (ICC) was computed separately for the pre- and post-consensus readings for *V*_pre_, *V*_mid_ and TVRR. Methodological details are given in Supplementary Method S3.

### Treatment protocol

Treatments followed harmonized protocols across the two centers, with minor institutional variation. Pelvic EBRT was delivered with 3D conformal RT to 50 Gy in 25 fractions (2.0 Gy per fraction; 1.8 Gy permitted). Central shielding was routinely introduced at 30–40 Gy when adequate BT coverage was anticipated on the mid-treatment MRI. Extended-field EBRT was used for para-aortic nodal involvement, in which nodes could receive an additional boost. BT was delivered to all patients, typically as four once-weekly fractions with intracavitary applicators by default. When coverage was anticipated to be inadequate, IC/IS or ISBT techniques were employed. At Center A, ISBT was delivered twice daily on consecutive days using a perineal interstitial template. Treatment planning was predominantly CT-based image-guided adaptive brachytherapy (IGABT), with a few early cases planned using orthogonal radiograph-based planning. MRI-based planning was not performed. Minimum doses to 90% of the high-risk clinical target volume (HR-CTV D90) for BT alone and the composite EBRT+BT (EBRT summed excluding central shielding) were reported following a multi-institutional prospective trial [7]. The doses are expressed as equivalent dose in 2-Gy fractions (EQD2) using the linear–quadratic model, with α/β = 10 Gy for the tumor. Concurrent chemotherapy consisted primarily of weekly cisplatin 40 mg/m^2^, with modifications permitted. Alternative platinum-based regimens were also allowed.

### Baseline, outcomes and statistical analysis

#### Baseline comparisons

Baseline characteristics, imaging and treatment variables were summarized overall and by center. Continuous variables are reported as medians with interquartile ranges (IQRs), and categorical variables as n (%). Between-center imbalances were quantified using absolute standardized mean differences (SMD); |SMD| ≥ 0.20 was considered notable. For analyses, ‘center’ was defined as the facility that delivered BT.

#### Outcomes

Overall survival (OS), progression-free survival (PFS) and local control (LC) were evaluated. Time zero for all outcomes was the start of RT. OS events were death from any cause, and PFS events were first progression (local/regional or distant) or death. For LC, the event was first documented as local failure, defined as recurrence or progression at the primary site. Deaths without prior local failure were treated as competing events.

#### Unadjusted outcomes by TVRR strata

Follow-up time was determined using the reverse Kaplan–Meier method. The survival function for OS and PFS was estimated using the Kaplan–Meier method. The cumulative incidence function (CIF) was estimated for LC and plotted as 1 − CIF. Patients were stratified *a priori* at TVRR cutoffs of 80%, 60% and 40% into four strata (≥80%, 60 to <80%, 40 to <60%, <40%). Curves are descriptive only. No between-stratum survival comparisons were performed. Separately, composite HR-CTV D90 (EQD2) was compared across strata by Kruskal–Wallis test with a Dunn–Holm test. Effect sizes (η^2^[H]) are reported. The correlation between TVRR and composite HR-CTV D90 was assessed using Spearman’s rank correlation coefficient.

### Adjusted time-to-event models (primary analyses)

A cross-walk of analysis-specific model specifications is shown in Supplementary Table S2. The proportional hazards assumption was assessed using Schoenfeld residuals for all time-to-event models.

#### OS and PFS

We fitted multivariable Cox proportional hazards models with TVRR entered via a penalized spline (linear effect reported per 10 percentage point [pp]; non-linearity tested at degrees of freedom [df] = 3). Age (per 10 years; continuous), nodal status (positive vs. negative; binary), histology (non-SCC vs. SCC; binary) and FIGO 2009 stage (III–IV vs. I–II; binary) were forced into the model because they are established prognostic factors in cervical cancer chemoradiation. Other prespecified candidates for covariates were overall treatment time (OTT; per 7 days; continuous), pre-treatment hemoglobin (Hb; per 1 g/dL; continuous), pre-treatment tumor volume (per 10 cm^3^; continuous), and chemotherapy category (cisplatin cumulative dose ≥200 mg/m^2^ vs. <200 mg/m^2^, or other; binary). Because the selection procedure operated on only four candidate variables, the risk of overfitting through variable selection was limited; model stability was further assessed through bootstrap optimism correction and internal-external cross-validation (IECV) (Section 2.10).

#### LC

LC was analyzed using a Fine–Gray subdistribution hazards model with death without prior local failure as the competing event. TVRR was entered via a penalized spline (linear effect per 10 pp; non-linearity tested at df = 3). Per a prespecified plan prioritizing covariates plausibly related to local control, FIGO 2009 stage (III–IV vs. I–II; binary), histology (non-SCC vs. SCC; binary), and pre-treatment tumor volume (per 10 cm^3^; continuous) were forced into the model. No data-driven selection was applied.

### Model validation

#### Internal validation

Discrimination was quantified using a Harrell-type C-index for the Cox models (OS and PFS) and for the Fine–Gray subdistribution hazard model (LC) [8]. Discrimination estimates were optimism-corrected by bootstrap resampling (B = 1000). Internal calibration used decile-grouped plots comparing model-predicted 60-month probabilities from the Cox baseline with Kaplan–Meier estimates for OS and PFS. Observed values were Kaplan–Meier 60-month estimates within each decile. Calibration was summarized by the linear calibration-in-the-large (intercept) and slope, estimated from an ordinary least-squares (OLS) regression of decile-grouped observed on predicted.

#### Internal-external cross-validation

IECV was conducted for OS and PFS. Leave-one-center-out validation (train on Center A, test on Center B; and vice versa) was performed using a core specification that included TVRR (continuous linear, per 10 pp) and the same covariates forced into the primary model (age, FIGO 2009 stage, nodal status and histology). On the held-out center, performance was evaluated using a Harrell-type C-index and 60-month calibration (quintile-grouped plots with OLS intercept/slope). Coefficients were not refit on the held-out data. For each split, 95% confidence intervals (CIs) for C-index and calibration parameters at 60 months were obtained by nonparametric bootstrap resampling of patients in the test cohort (B = 1000).

### Secondary/robustness analyses

#### Effect modification by center

For OS and PFS, Cox models including a TVRR×center interaction were fitted, entering TVRR as a continuous linear term with effects reported per 10 pp, and adjusting for age, FIGO 2009 stage, nodal status and histology. Center-specific hazard ratios per 10-pp increase in TVRR with 95% CIs were derived from linear contrasts. The interaction was tested by a likelihood-ratio test.

#### Sensitivity analysis

Sensitivity analyses used the implementation (linear) specification, replacing the TVRR spline with a linear term per 10 pp while retaining the final covariates from the full specification. Two exploratory subsets were examined: (i) a combined MRI-timing subset (pre-treatment MRI ≤28 days before RT start and mid-treatment MRI at 26–30 Gy), and (ii) squamous cell carcinoma (SCC) treated with weekly concurrent cisplatin and CT-based IGABT. Effect modification was assessed via TVRR×subset interactions contrasting each subset with its complement (remainder) within the full analysis population for OS and PFS (likelihood-ratio test). Additionally, to assess whether TVRR is independent of dose adequacy, the implementation model was refitted with composite HR-CTV D90 EQD2 (per 10 Gy; continuous) forced as an additional covariate.

### Software, reproducibility and missing data

To enhance transparency and reproducibility, coefficients and 12/36/60-month baseline survival probabilities for the linear implementation model are given (Supplementary Table S3A–C). A standalone browser-based research-use calculator implementing this model is available in the Supplementary Web Calculator (https://t-saito-rad.github.io/tvrr-calculator/). All analyses were performed in R (ver. 4.4.3) [9], using the irr (ver. 0.84.1) [10], survival (ver. 3.8–3) [11] and tidycmprsk (ver. 1.1.0) [12] packages. Two-sided *P* < 0.05 was considered statistically significant. No imputation was required. Complete-case analyses coincided with the full analytic cohort.

## RESULTS

### Cohort assembly

**Fig. 1:**
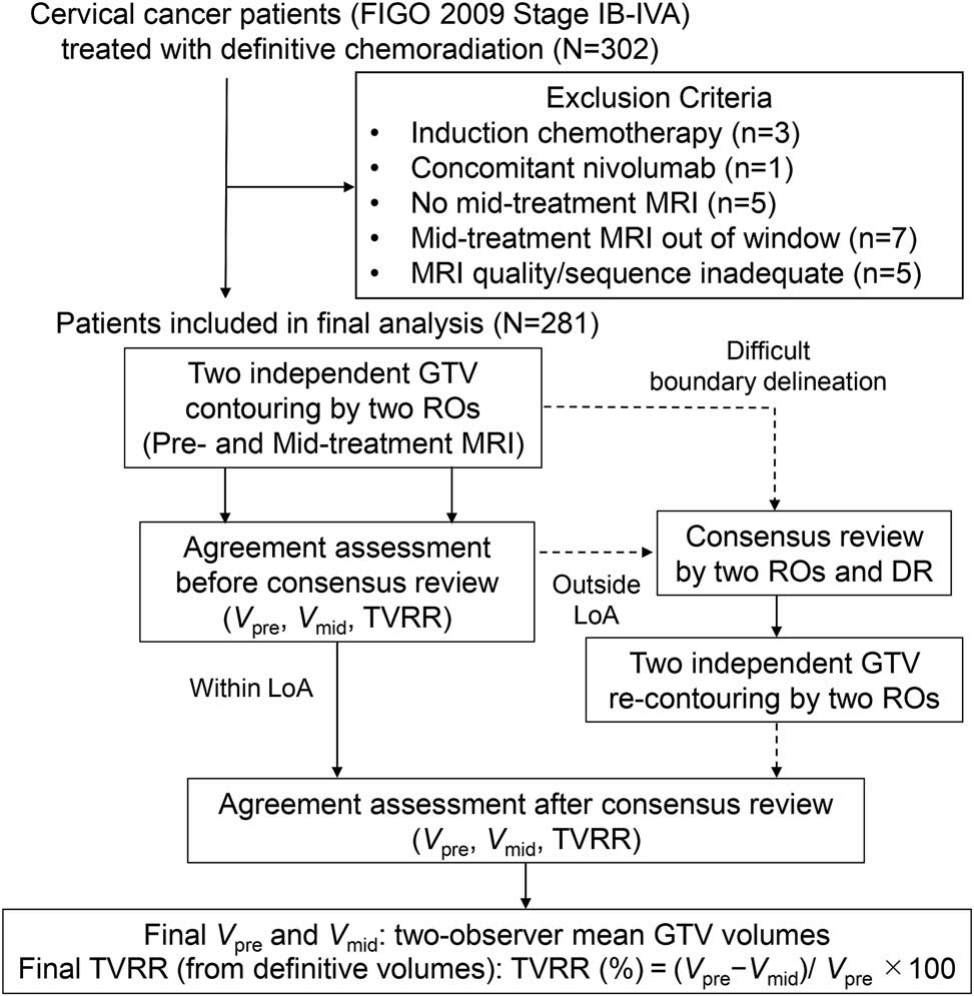
Cohort assembly and contouring workflow. Abbreviations: FIGO, International Federation of Gynecology and Obstetrics; MRI, magnetic resonance imaging; GTV, gross tumor volume; RO, radiation oncologist, *V*_pre_, pre-treatment volume; *V*_mid_, mid-treatment volume; TVRR, tumor volume reduction rate; DR, diagnostic radiologist; LoA, limits of agreement.

Of a total of 302 consecutive patients with FIGO 2009 stage IB–IVA cervical cancer treated with definitive chemoradiation, 281 met the eligibility criteria and comprised the analytic cohort. Reasons for exclusion are shown in Fig. 1. Information relevant to the generalizability of the findings across the two centers is provided in Supplementary Table S1.

### Baseline characteristics, imaging and treatment

Baseline characteristics, imaging and treatment are summarized in Table 1. Unless otherwise specified, values are median (IQR) or n (%). The cohort was typical of locally advanced cervical cancer: median age 59 years, performance status 0–1 98%, SCC histology 85%, FIGO 2009 stage III–IV 60% and nodal metastasis 56%. *V*_pre_ and *V*_mid_ were 50.5 (27.0–90.1) cm^3^ and 17.2 (7.8–31.8) cm^3^, respectively. Pre-treatment MRI was acquired 20 (13–25) days before the start of RT; mid-treatment MRI was acquired at 28 (26–30) Gy. TVRR was 66.6% (50.3–79.0%); the full distribution is shown in Supplementary Fig. S1. An additional nodal boost was delivered in 76 patients (27%), with a median dose of 6 (6–10) Gy. An interstitial technique (IC/IS or ISBT) was used in 22% of patients, and HR-CTV D90 was unavailable in 15/281 (5%) due to orthogonal radiograph-based planning. Concurrent chemotherapy was predominantly weekly cisplatin (95%). Among patients receiving cisplatin, the median number of weekly cycles was 5 (4–6), and 68% received ≥5 cycles. By center (Center A, *n* = 160; Center B, *n* = 121), multiple imbalances exceeded |SMD| ≥ 0.20 for disease burden, volumetrics, BT technique/dosimetry, systemic therapy and OTT, while age, performance status, hemoglobin and MRI timing were comparable (Table 1).

**Table 1:** Baseline characteristics, imaging and treatment. Unless otherwise specified, values are median (IQR) or n (%)

Item	Category	All (*n* = 281)	Center A (*n* = 160)	Center B (*n* = 121)	|SMD|
Median follow-up period, months	—	69.6 (54.2–87.3)	69.6 (54.2–86.0)	69.8 (54.2–96.7)	—
Patients & tumors					
Age, years	—	59 (49–66)	59 (49–67)	58 (49–65)	0.072
Performance status	0–1	274 (98)	157 (98)	117 (97)	0.090
	2	7 (2)	3 (2)	4 (3)	
Histology	SCC	238 (85)	124 (78)	114 (94)	0.858
	Non-SCC	43 (15)	36 (22)	7 (6)	
FIGO 2009 stage	I–II	111 (40)	34 (21)	77 (64)	0.413
	III–IV	170 (60)	126 (79)	44 (36)	
Nodal status	Negative	125 (44)	57 (36)	68 (56)	0.480
	Positive	156 (56)	103 (64)	53 (44)	
Pre-treatment hemoglobin, g/dl	—	11.8 (10.5–12.8)	11.9 (10.4–12.8)	11.7 (10.6–12.9)	0.065
MRI timing & volumetry					
Pre-treatment volume, cm^3^	—	50.5 (27.0–90.1)	59.9 (30.3–98.0)	44.7 (23.9–75.2)	0.340
Pre-treatment MRI to RT start, days	—	20 (13–25)	20 (15–24)	19 (12–28)	0.016
Mid-treatment volume, cm^3^	—	17.2 (7.8–31.8)	20.8 (11.5–39.9)	12.6 (4.9–22.7)	0.517
Dose at mid‑treatment MRI, Gy	—	28 (26–30)	28 (26–30)	30 (28–30)	0.101
RT start to mid-treatment MRI, days	—	20 (17–22)	20 (17–22)	20 (18–22)	0.069
Tumor volume reduction rate, %	—	66.6 (50.3–79.0)	61.9 (46.1–72.2)	73.8 (58.7–81.5)	0.474
Treatment details					
Brachytherapy technique	Intracavitary	219 (78)	108 (67)	111 (92)	0.631
	IC/IS or ISBT	62 (22)	52 (33)	10 (8)	
HR-CTV D90 EQD2 ^*^ (BT component), Gy	—	37.9 (33.4–42.6)	36.3 (32.7–40.1)	40.1 (36.2–46.6)	0.508
HR-CTV D90 EQD2 ^*^ (composite EBRT + BT), Gy	—	71.5 (67.5–76.4)	70.0 (66.3–72.9)	75.4 (69.4–80.6)	0.664
Concurrent chemotherapy	Weekly cisplatin	266 (95)	145 (91)	121 (100)	0.444
	Other regimen	15 (5)	15 (9)	0 (0)	
Overall treatment time, days	—	46 (44–51)	45 (43–49)	49 (44–51)	0.535

Abbreviations: SMD, standardized mean difference; SCC, squamous cell carcinoma; FIGO, International Federation of Gynecology and Obstetrics; MRI, magnetic resonance imaging; RT, radiotherapy; IC/IS, intracavitary and interstitial brachytherapy; ISBT, interstitial brachytherapy; HR-CTV D90, Minimum dose to 90% of the high-risk clinical target volume; EQD2, equivalent dose in 2-Gy fractions; BT, brachytherapy; EBRT, external beam radiotherapy; IQR, interquartile range

^*^HR-CTV D90 was unavailable in 15/281 cases (5%) due to orthogonal radiograph-based planning.

### Interobserver agreement

**Fig. 2:**
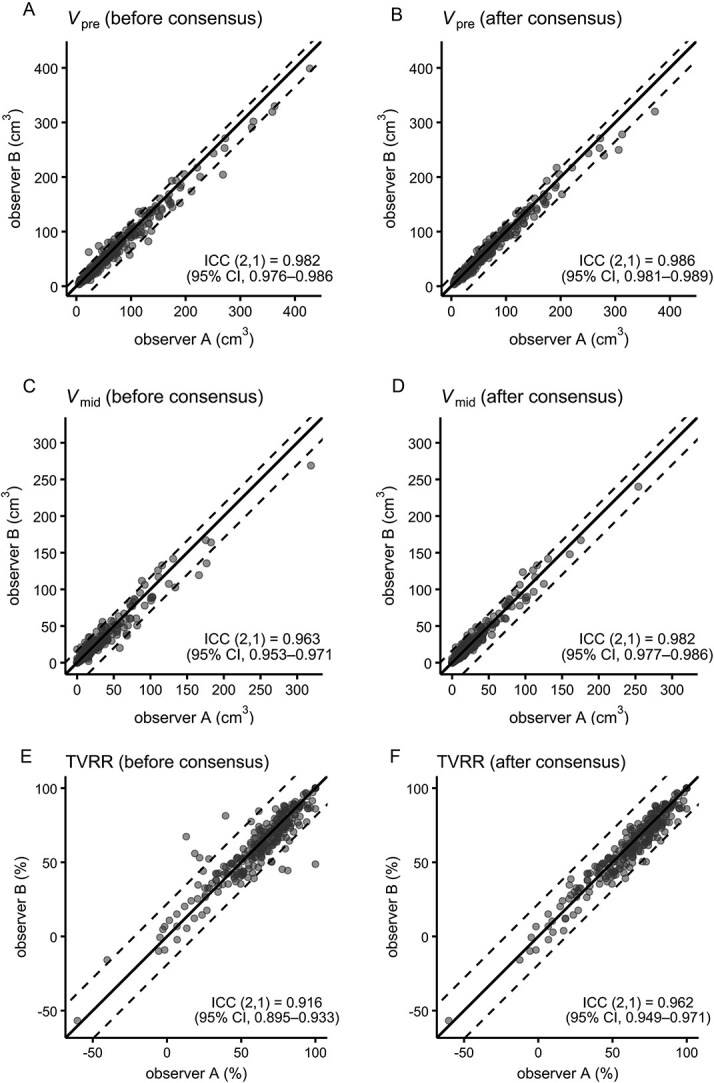
Interobserver agreement before and after consensus review. Scatter plots compare observer A vs. observer B for (A) *V*_pre_ before consensus; (B) *V*_pre_ after consensus; (C) *V*_mid_ before consensus; (D) *V*_mid_ after consensus; (E) TVRR before consensus; (F) TVRR after consensus. Dotted lines indicate the prespecified non-parametric LoA used to triage cases for consensus review. In the post-consensus panels (B, D, F), the same pre-consensus LoA are overlaid. Abbreviations: *V*_pre_, pre-treatment volume; *V*_mid_, mid-treatment volume; TVRR, tumor volume reduction rate; LoA, limits of agreement.

Consensus review was triggered in 39/281 cases (13.9%) under the prespecified rules. Interobserver agreement was high and improved after the consensus procedure (Fig. 2). ICC increased from 0.982 to 0.986 for *V*_pre_, from 0.963 to 0.982 for *V*_mid_, and from 0.916 to 0.962 for TVRR.

### Unadjusted outcomes by TVRR strata

**Fig. 3:**
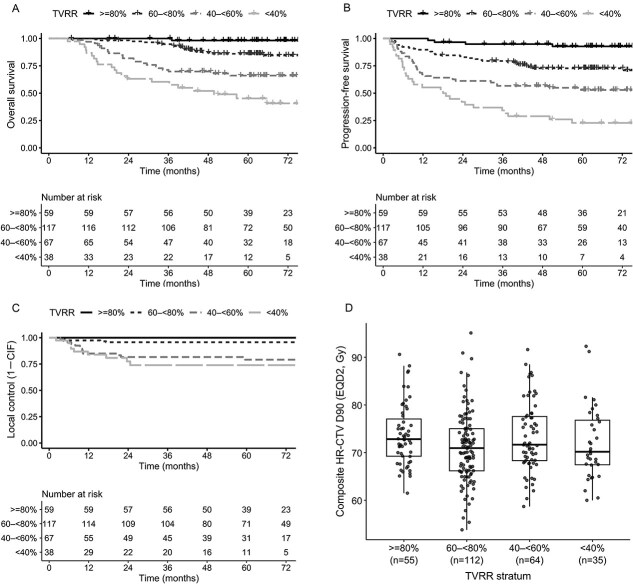
Unadjusted outcomes by prespecified TVRR strata. Kaplan–Meier curves for (A) overall survival and (B) progression-free survival, and (C) 1 − CIF for local control, each stratified by TVRR (≥80%, 60–<80%, 40–<60%, <40%). (D) Distribution of composite HR-CTV D90 (EQD2) across TVRR strata; Kruskal–Wallis *P* = 0.060; η^2^[H] = 0.017; all Dunn–Holm pairwise comparisons non-significant. Composite HR-CTV D90 was missing for 15 patients. Abbreviations: TVRR, tumor volume reduction rate; CIF, cumulative incidence function; HR-CTV D90, minimum dose to 90% of the high-risk clinical target volume; EQD2, equivalent dose in 2-Gy fractions.

In a median follow-up period of 69.6 months (IQR, 54.2–87.3 months), there were 65 deaths (56 cancer-specific, nine other-cause), 97 PFS events and 27 local failures. By center (Center A vs. Center B), these data were: cancer-specific deaths 43 vs. 13; other-cause deaths 2 vs. 7; PFS events 62 vs. 35; and local failures 18 vs. 9. Kaplan–Meier curves (OS, PFS) and 1 − CIF curves for LC (death without prior local failure as the competing event) separated across prespecified TVRR strata (Fig. 3A–C). For the entire cohort, the 5-year OS, PFS and LC were 78.3%, 65.3% and 90.2%. Composite HR-CTV D90 did not differ significantly across strata (Kruskal–Wallis *P* = 0.060; η^2^[H] = 0.017; all Dunn–Holm pairwise comparisons not significant) (Fig. 3D). At the individual patient level, TVRR and composite HR-CTV D90 showed no significant correlation (Spearman ρ = 0.089, *P* = 0.149; Supplementary Fig. S3).

### Adjusted associations of TVRR with oncologic outcomes

**Fig. 4:**
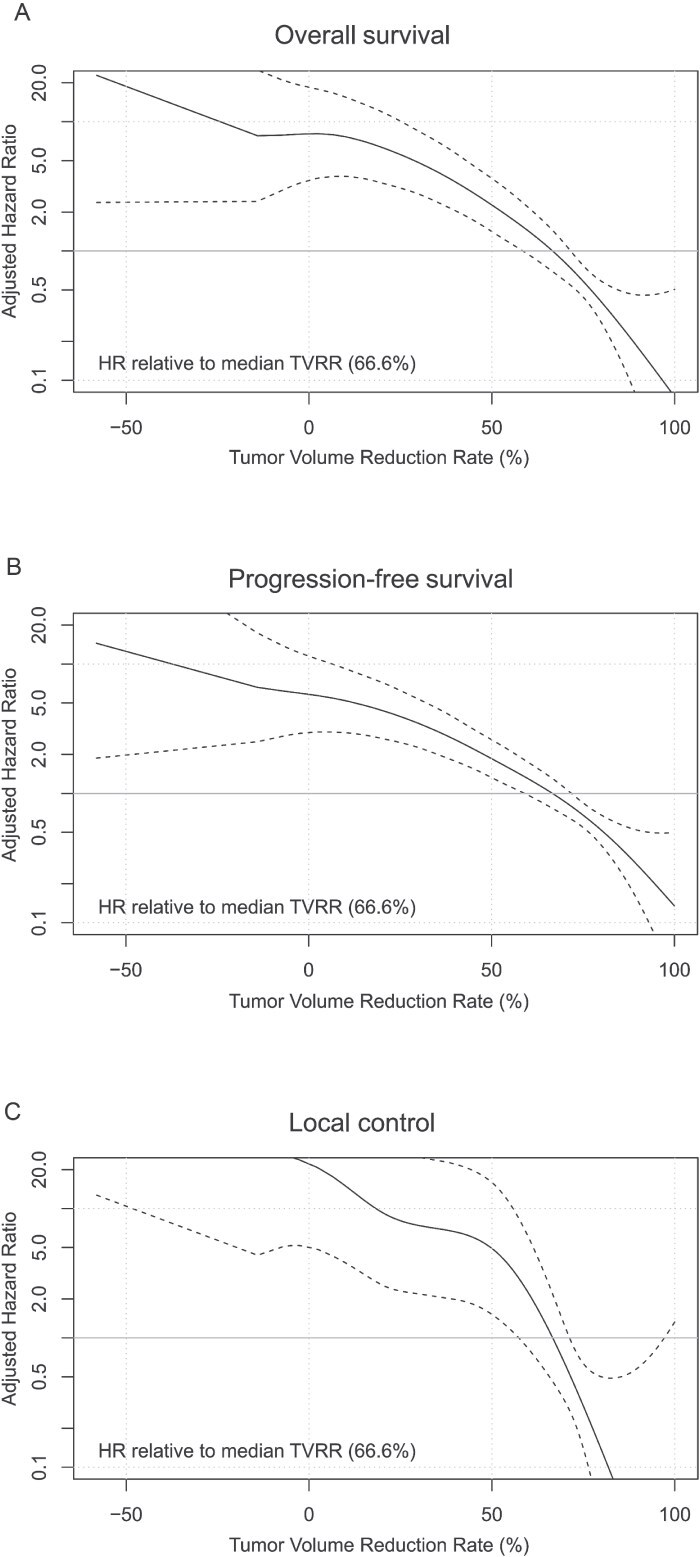
Adjusted associations of TVRR and model performance. (A) Cox model for OS with TVRR modeled with a penalized spline; HRs are shown relative to the cohort median TVRR (66.6%) with 95% CIs (y-axis on a log scale; horizontal line at HR = 1). (B) Cox model for PFS; (C) Fine–Gray model for LC; in all three, tests for nonlinearity were non-significant. Abbreviations: TVRR, tumor volume reduction rate; OS, overall survival; HR, hazard ratio; CI, confidence interval; PFS, progression-free survival; LC, local control.

In multivariable analyses, higher TVRR remained independently associated with better outcomes across endpoints. Full estimates are shown in Table 2 and visualized in Fig. 4. For OS and PFS, AIC-based selection retained pre-treatment Hb for OS and pre-treatment Hb and OTT for PFS. Chemotherapy category was not retained for any endpoint. There were no missing covariate values in the primary models. The proportional hazards assumption was satisfied for TVRR across all models (Schoenfeld residuals, *P* ≥ 0.67; global test, *P* ≥ 0.20). For nodal status in the PFS model, evidence of non-proportional hazards was observed (*P* = 0.004); its hazard ratio should therefore be interpreted as an average effect over time.

**Table 2:** Multivariable time‑to‑event models for clinical outcomes with TVRR

Item	Overall survival (Events 65/281)		Progression-free survival (Events 97/281)		Local control (Events 27/281)	
Variables (definition)	HR (95% CI)	Wald χ^2^ (*P*-value)	HR (95% CI)	Wald χ^2^ (*P*-value)	sHR (95% CI)	Wald χ^2^ (*P*-value)
TVRR—linear term (per 10-pp increase)	0.71 (0.64–0.79)	42.7 (<0.001)	0.74 (0.68–0.81)	48.4 (<0.001)	0.68 (0.58–0.80)	21.8 (<0.001)
TVRR—nonlinear component (degrees of freedom = 3)	—	6.1 (0.109)	—	4.3 (0.229)	—	5.2 (0.164)
Age (per 10 years increase)	1.20 (0.95–1.52)	2.4 (0.122)	1.05 (0.88–1.26)	0.3 (0.576)	—	—
Histology (non-SCC vs. SCC (ref))	1.45 (0.79–2.65)	1.5 (0.228)	2.03 (1.26–3.27)	8.5 (0.004)	0.31 (0.08–1.13)	3.2 (0.075)
FIGO 2009 stage (Stage III–IV vs. I–II (ref))	1.42 (0.73–2.78)	1.1 (0.301)	1.26 (0.77–2.06)	0.8 (0.366)	1.08 (0.34–3.38)	0.0 (0.897)
Nodal status (positive vs. negative (ref))	1.99 (1.13–3.50)	5.7 (0.017)	2.36 (1.48–3.75)	13.2 (<0.001)	—	—
Pre-treatment hemoglobin (per 1 g/dL increase)	0.87 (0.75–1.01)	3.5 (0.062)	0.91 (0.81–1.03)	2.2 (0.135)	—	—
Overall treatment time (per 7-day increase)	—	—	1.27 (1.02–1.59)	4.6 (0.033)	—	—
Pre-treatment tumor volume (per 10 cm^3^ increase)	—	—	—	—	1.06 (0.99–1.13)	2.7 (0.098)
Model information	Likelihood–ratio χ^2^ = 82 (df = 9, *P* < 0.001)		Likelihood–ratio χ^2^ = 100 (df = 10, *P* < 0.001)		Global Wald χ^2^ = 44 (df = 7, *P* < 0.001)	
Internal discrimination (optimism-corrected C-index)	0.74		0.73		0.81	
Internal calibration at 60 months	intercept = 0.015 Slope = 0.985		intercept = 0.021 Slope = 0.974		—	

Multivariable Cox models were fitted for OS and PFS; a Fine–Gray subdistribution hazards model was used for LC (death without prior local failure as a competing event). An em dash (—) indicates that the covariate was not included in the final model for that endpoint.

Abbreviations: TVRR, tumor volume reduction rate; HR, hazard ratio; sHR, subdistribution hazard ratio; CI, confidence interval; pp, percentage points; SCC, squamous cell carcinoma; FIGO, International Federation of Gynecology and Obstetrics.

Modeled with a penalized spline, the linear TVRR component (per 10 pp) was associated with lower hazards of death and disease progression: OS hazard ratio (HR) 0.71 (95% CI 0.64–0.79, *P* < 0.001), PFS HR 0.74 (0.68–0.81, *P* < 0.001) and LC subdistribution HR 0.68 (0.58–0.80, *P* < 0.001). Non-linear components were not significant (*P* = 0.11, 0.23, 0.16 for OS, PFS, LC, respectively). TVRR had the largest Wald χ^2^ among retained covariates (Table 2). After AIC-based selection, nodal positivity was independently associated with worse OS and PFS, whereas non-SCC histology and longer OTT were associated with inferior PFS. The other factors were not independently significant in the full specification.

In internal validation, the bootstrap optimism-corrected C-index was 0.74 for OS, 0.73 for PFS and 0.81 for LC. Apparent decile-grouped calibration at 60 months (Cox baseline-predicted vs. Kaplan–Meier estimates) yielded: for OS, intercept 0.015 and slope 0.985; and for PFS, intercept 0.021 and slope 0.974. Calibration plots at 60 months for OS and PFS are shown in Supplementary Fig. S2.

**Fig. 5:**
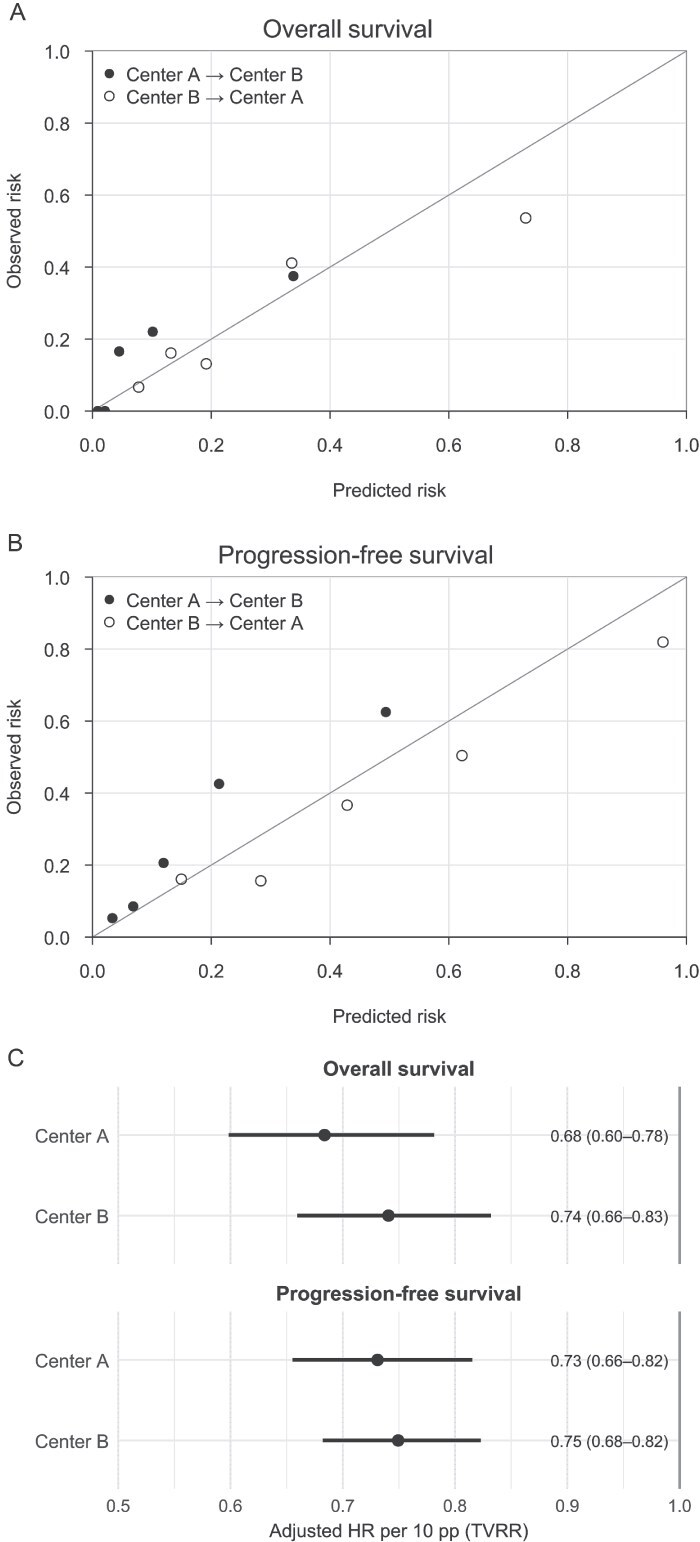
Model performance. (A–B) IECV calibration at 60 months for OS (A) and PFS (B), shown for both directions (train Center A→test Center B; train Center B→test Center A). (C) Center-specific TVRR effect (HR per 10-percentage-point increase) from the interaction model; points indicate HRs and bars 95% CIs. Abbreviations: TVRR, tumor volume reduction rate; OS, overall survival; PFS, progression-free survival; LC, local control; HR, hazard ratio; CI, confidence interval; IECV, internal-external cross-validation.

IECV discrimination estimates in both directions were as follows. Training on the Center A cohort (*n* = 160; OS events = 45; PFS events = 62) and testing on the Center B cohort (*n* = 121; OS events = 20; PFS events = 35) yielded a C-index of 0.79 (95% CI 0.62–0.96) for OS and 0.79 (0.66–0.91) for PFS. At 60 months, calibration (quintile-grouped observed vs. predicted) for this split was: OS, intercept 0.044 (−0.021–0.112) and slope 1.055 (0.441–1.798); PFS, intercept 0.045 (−0.051–0.127) and slope 1.260 (0.869–1.784). Training on the Center B cohort and testing on the Center A cohort yielded a C-index of 0.72 (0.57–0.87) for OS and 0.73 (0.61–0.85) for PFS. At 60 months, calibration for this split was: OS, intercept 0.053 (−0.044–0.165) and slope 0.720 (0.337–1.118); and PFS, intercept −0.019 (−0.130–0.115) and slope 0.860 (0.598–1.072). Calibrations at 60 months for the IECV splits are shown in Fig. 5A–B.

In the interaction model (TVRR per 10 pp × center; adjusted for age, FIGO 2009 stage, nodal status and histology), the center-specific HRs per 10-pp increase in TVRR were Center A 0.68 (95% CI 0.60–0.78), Center B 0.74 (0.66–0.83) and TVRR×center interaction *P* = 0.371 for OS; and Center A 0.73 (0.66–0.82), Center B 0.75 (0.68–0.82) and interaction *P* = 0.731 for PFS (Fig. 5C).

Sensitivity analyses are shown in Supplementary Table S4 (panels A and B). Across both subsets, there was no evidence of heterogeneity in the TVRR-outcome association (TVRR×subset interaction) for OS and PFS (panel C). When composite HR-CTV D90 was added as a covariate, the TVRR effect was unchanged and D90 was not significant (panel D; 15 patients lacking D90 due to 2D planning were excluded). For clinical interpretability, representative predicted 5-year absolute risks for OS and PFS according to TVRR are shown in Supplementary Table S3D.

## DISCUSSION

This study showed that mid-treatment MRI-based TVRR is strongly linearly associated with all analyzed outcomes. Per 10-pp increase in TVRR, hazards were 26–32% lower across endpoints, with no material evidence of nonlinearity over the observed range. Within each adjusted model, TVRR contributed most prognostic information (highest Wald χ^2^), exceeding that of established covariates (e.g. nodal status, histology, FIGO 2009 stage). Internal validation showed moderate-to-high discrimination and close 60-month calibration, and findings were consistent across sensitivity subsets. In IECV, held-out discrimination was similar irrespective of training direction, and 60-month calibration intercepts were near zero. However, CIs for the C-indexes and calibration parameters were wide, thus the IECV results primarily support consistency of model performance between centers and the absence of gross miscalibration, rather than precise center-specific estimates. Calibration slopes differed by split: >1 when trained on Center A and < 1 when trained on Center B. This pattern is consistent with the case mix at the two centers: Center A contributed more advanced-stage and non-SCC cases, whereas Center B had more early-stage and SCC cases and fewer high-risk cases and OS events. As reported in Section 3.4, other-cause deaths were more frequent at Center B and may have contributed to between-center calibration differences. These findings support simple site-specific recalibration for transportability. Even so, the estimated effect of a 10-pp increase in TVRR was essentially the same in Centers A and B, and formal interaction testing gave no evidence of effect modification by center (Fig. 5C). Taken together, these results support TVRR as a promising, threshold-free prognostic biomarker, warranting external validation in independent cohorts.

TVRR is a continuous on-treatment biomarker integrating two processes: clearance of non-viable cells, summarized as the dead-cell-resolving half-life (T_1/2_), and intrinsic radiosensitivity proxied by the 2-Gy surviving fraction (SF2) [13, 14]. In kinetic-model analyses of cervical cancer, shorter T_1/2_ predicts better outcomes; in multivariable models T_1/2_ was independently associated with outcomes, whereas conventional clinicopathologic factors contributed less and were not independently significant [14]. Radiosensitivity worsens under hypoxia (higher SF2), and longitudinal dynamic contrast-enhanced MRI perfusion patterns capture oxygenation dynamics: high baseline perfusion or early reoxygenation is associated with better outcomes, whereas persistently low perfusion is associated with worse outcomes [15]. Consistent with TVRR reflecting tumor biology rather than dose adequacy, the TVRR effect was unchanged after adjustment for composite HR-CTV D90 (Supplementary Table S4D). In the scatter plot (Supplementary Fig. S3), local failures appeared to cluster among low-TVRR cases regardless of D90, while high-TVRR cases appeared to remain locally controlled even at lower D90 values. Low-TVRR cases also had a high rate of competing deaths without documented local failure, indicating that low TVRR signals poor prognosis beyond LC. These findings support a role for TVRR in guiding risk-adapted treatment strategies, including both BT optimization and systemic intensification, pending prospective validation.

Oncology is moving from fixed risk categories to continuous, response-based prediction. In cervical cancer, the HR-CTV volume at the time of BT is a key determinant of LC and is used routinely to guide technique selection, with larger targets typically prompting combined IC/IS approaches to secure adequate coverage [16]. However, interobserver variability is higher with CT than with MRI [4], raising concerns about using HR-CTV as a dose-defining metric in CT-based BT. Contemporary multisociety guidance recommends obtaining a pre-BT MRI even in CT-based BT programs [17]. Our results show that a pragmatic mid-treatment MRI protocol yields TVRR, a response measure strongly associated with oncologic outcomes. With standardized contouring, two radiation oncologists achieved high reproducibility for tumor volumes and TVRR, aligning with prior literature [18]. These data support routine mid-treatment MRI to quantify response, especially in settings in which MRI-guided BT is not feasible, such as many Asian centers where CT-based BT remains predominant [19].

In prior studies using threshold-based analyses, later assessments near 45–50 Gy consistently yielded stronger associations [20]. However, the same data show that regression ratios converge toward near-complete regression by that timepoint (mean residual volume ratio 18% at 45–50 Gy vs. 56% at 20–25 Gy) [20]. When TVRR is modeled as a continuous variable, such distributional compression may limit prognostic discrimination through a ceiling effect. In contrast, early measurements at 20–25 Gy often show weaker discrimination, likely because morphologic regression is still emerging at that time [20]. In our cohort, the mid-treatment MRI was obtained at a median of 28 (IQR 26–30) Gy, at about 20 (IQR 17–22) days from RT start, corresponding to weeks 2.5 to 3. The median TVRR of 66.6% (IQR 50.3–79.0%) preserved sufficient inter-patient heterogeneity, and TVRR was linearly associated with all endpoints with a large effect size. Although we do not claim that our imaging window is optimal, it offers two pragmatic benefits compared with the pre-BT window. First, residual volume is generally sufficient to support more reliable contouring and quantitative TVRR estimation, with less susceptibility to floor and partial-volume effects. By the pre-BT window, regression is often profound and small foci may be indistinct. Second, an earlier read-out enables risk-adapted decisions during EBRT, rather than after it. In particular, earlier prognostication can anticipate the need for an interstitial technique and, when complex implants are expected, permit timely referral to centers with greater BT expertise, as is the practice in this healthcare region. A current barrier to routine TVRR assessment is the contouring workload, but advances in AI-based auto-segmentation are rapidly reducing this burden and may facilitate prospective implementation. Prospective studies incorporating scheduled TVRR assessment could refine risk estimation and support the development of risk-adapted individualized treatment.

The most promising use of TVRR for personalization is de-escalation in good responders. In EMBRACE-I, 5-year LC was 92% while severe late morbidity (grade ≥ 3) remained at 18% [21]. EMBRACE-II operationalizes dose modulation, permitting HR-CTV D90 ≥ 85 Gy for HR-CTV <30 cm^3^ while generally targeting 90 to <95 Gy [22]. An international multi-institutional retrospective study from Asia likewise suggested a low-risk subgroup that is potentially curable with lower doses [23]. However, these de-escalation schemes typically convert continuous predictors into categories using fixed thresholds. Ideally, the optimal dose should vary smoothly and be estimated per patient by fitting a reliable continuous predictor. TVRR is a biologically grounded, promising candidate for such patient-specific optimization. Conversely, in our cohort, lower TVRR was associated with inferior LC and worse OS and PFS, suggesting that escalation may need to extend beyond local dose modification to include systemic intensification. Recent phase 3 data show that adding ICI to chemoradiation improves outcomes [24]. However, whether the benefit of ICI accrues uniformly across patients remains uncertain. Because low TVRR is associated with poorer prognosis, such patients may have greater headroom for absolute benefit from adding ICI, whereas patients with high TVRR, who already have favorable outcomes, may have limited room for further gain. This is a hypothesis-generating idea that requires prospective evaluation.

As limitations, this two-center, retrospective study was not fully harmonized across time or between institutions. Both centers were located within a single healthcare region; referral patterns between them may have introduced selection bias, and generalizability to different practice settings remains uncertain. The timing of mid-treatment MRI varied within a prespecified window, and treatment techniques were not strictly uniform. Furthermore, mid-treatment MRI in this study was obtained at ~30 Gy, earlier than the 40–45 Gy timepoint used in many contemporary practices; findings may not directly generalize to assessments performed in the pre-BT window. Our composite HR-CTV D90 (EQD2) values likely underestimate the central target dose because central shielding was used. Phantom and planning studies indicate that, when BT is combined with central shielding, several Gy (EQD2) may be delivered to the central target [25]. Indeed, a clinical study using the same central-shielding-based approach found that composite HR-CTV D90 was not associated with LC [26]. Whether these findings generalize to settings in which central shielding is not used remains to be verified. The modest number of local failures also limited the precision of LC estimates, and analyses for LC should therefore be regarded as exploratory. We could not secure an independent dataset that met our prespecified mid-treatment MRI timing and protocol requirements, so external validation was not feasible.

In conclusion, mid-treatment MRI-based TVRR provides a strong, approximately linear prognostic signal across OS, PFS and LC. In multivariable models, each 10-pp increase corresponded to ~26–32% lower hazards, and TVRR contributed the greatest prognostic information among evaluated covariates. Modeling TVRR as a continuous predictor enables threshold-free, calibrated risk estimation. In two-center internal–external cross-validation, discrimination was similar between centers and 60-month calibration did not show major miscalibration. These findings support the use of continuous TVRR in the design of risk-adapted escalation/de-escalation trials. Independent external validation is required before clinical implementation.

## Supplementary Material

Supplementary_material_rrag043

## Data Availability

Patient-level data are not publicly available due to ethical and legal restrictions under our IRB approvals and opt-out consent framework. Model coefficients and 12/36/60-month baseline survival landmarks required for individual predictions are provided in Supplementary Table S3. In addition, a standalone browser-based research-use calculator implementing the linear OS and PFS models is provided as Supplementary Web Calculator (https://t-saito-rad.github.io/tvrr-calculator/). This tool is intended solely for research and educational use and is not intended for clinical decision-making.

## DECLARATIONS

Ethics approval and consent to participate: This study was approved by the ethics committee of Tsukuba Clinical Research & Development Organization (Registration number: R05-088) and the Institutional Review Board of Ibaraki Prefectural Central Hospital (Registration number: 1699).

## PRESENTATION AT A CONFERENCE

This work was presented at The 38th Annual Meeting of the Japanese Society for Radiation Oncology (JASTRO 2025), Tokyo, Japan, November 27–29, 2025.
