# Supplementary material for: Mid-treatment MRI-based tumor volume reduction rate as a continuous prognostic factor after chemoradiation for cervical cancer: development and two-center internal–external validation
Source: J Radiat Res. 2026 Jun 25;67(4):634–47. doi: 10.1093/jrr/rrag043 (PMC13400566; doi:10.1093/jrr/rrag043)
Supplement: Supplementary_material_rrag043 [file supplementary_material_rrag043.zip › Supplementary_revised(clean)_rrag043.docx]

Abbreviations (supplement): ESUR, European Society of Urogenital Radiology; FSE, fast spin echo, DWI, diffusion weighted image; ADC, apparent diffusion coefficient, RT, radiotherapy, EBRT, external beam radiotherapy; GTV, gross tumor volume; T2WI, T2-weighted imaging; PMI, parametrial invasion; TVRR, tumor volume reduction rate; ICC, intraclass correlation coefficient; IQR, interquartile range; OS, overall survival; PFS, progression-free survival; SCC, squamous cell carcinoma; HR, hazard ratio; LC, local control

Supplementary Methods

S1. Imaging acquisition and adequacy (full criteria). Image-quality guidance was based on ESUR MRI guidelines [S1, S2].

Eligibility: Required and Allowed

- Sequences/planes. Two-dimensional T2-weighted FSE in two orthogonal planes: one axial-oblique (preferred) or conventional axial plane, and a second orthogonal plane: sagittal preferred, with coronal accepted if sagittal unavailable. T2 fat-sat was not used; DWI/ADC, when available, were reviewed qualitatively and did not determine eligibility.
- Coverage and artifacts. Field-of-view sufficient to include uterus and upper vagina; no severe motion/susceptibility artifacts that preclude tumor-stroma margin delineation.
- Hardware. 1.5 T or 3 T scanners.
- Slice thickness and gap. Preferred ≤4 mm (ESUR-2021), but ≤5 mm permitted (ESUR-2011 allowed 3–6 mm); inter-slice gap ≤1 mm was accepted (equivalent to ≤25% for 4-5 mm slices).
- Timing. Pre-treatment MRI generally within 4 weeks before RT start (eligibility up to 8 weeks); mid-treatment MRI during EBRT at approximately 30 Gy (permitted window 24–36 Gy).

S2. GTV delineation (full rules). Delineation followed a prespecified protocol derived from the ESUR MRI guidelines, as in S1, for MRI-based target definition in cervical cancer [S1, S2].

- Working series. For each patient and time point, one working series for volumetry was fixed *a priori* and used by both observers (axial-oblique prescribed perpendicular to the endocervical canal preferred). When axial-oblique was unavailable or suboptimal, conventional axial or sagittal T2WI was used as the working series. In each patient, the same working plane was used across time points. The orthogonal plane served for cross-reference. DWI/ADC were used qualitatively for cross-reference only; isolated DWI hyperintensity without a T2 correlate did not alter boundaries.
- Anatomical definition. GTV was the macroscopic tumor showing intermediate-to-high T2 signal relative to cervical stroma with definable margins. Parametrial invasion (PMI) was contoured only when full-thickness stromal invasion co-occurred with a spiculated or nodular tumor-parametrial interface and/or encasement of parametrial vessels; preservation of the outer low-signal stromal rim excluded PMI. Vaginal involvement was included only when contiguous T2-hyperintense wall thickening crossed the fornix with disruption of the low-signal submucosal line. Internal necrosis/cysts within tumor were included within the outer boundary, whereas isolated edema without a convincing tumor rim were excluded.
- Paired-timepoint workflow (longitudinal consistency). To ensure that the same lesion extent was delineated across time points and to minimize intraobserver variability, pre- and mid-treatment MRIs were loaded side-by-side and contoured sequentially in one sitting when feasible, using the same working plane. Each contour was determined on images at that time point. The other time point served only to confirm anatomic correspondence and avoid inclusion of non-tumoral tissue.

S3. Interobserver agreement and consensus workflow (full details)

- Subjective triage. If either observer judged the tumor boundary difficult to delineate, the case was routed to consensus review. Both observers still completed their independent contours, and these were included in the agreement analyses; consensus review was performed regardless of the measured agreement.
- Objective triage. For each case, the difference between the two observers was calculated for pre- and mid-treatment tumor volumes (cm³) and for TVRR (percentage points). The nonparametric limits of agreement were the empirical bounds enclosing the central 95% of these differences; any case outside these bounds proceeded to consensus review.
- Agreement calculations. Interobserver agreement was summarized by the intraclass correlation coefficient (ICC[2,1], two-way random, absolute agreement) computed on the pre-consensus contours and again after consensus. Visualization used observer-vs-observer scatter plots with equal axes and an identity line; each plot was annotated with nonparametric 95% limits of agreement derived from the distribution of interobserver differences (volumes in cm³; TVRR in percentage points).
- Final volume determination. For each case and time point, the definitive volume was the arithmetic mean of the two observers’ contours.

- No-consensus cases: the mean of the two initial, independent contours.

- Consensus cases: after a joint discussion, each observer independently revised their contour; the definitive volume was the mean of these post-consensus contours.

- The final TVRR was then recomputed from the definitive pre- and mid-treatment tumor volumes for all cases (negative values allowed).

Supplementary References

S1. Balleyguier C, Sala E, Da Cunha T et al. Staging of uterine cervical cancer with MRI: guidelines of the European Society of Urogenital Radiology. Eur Radiol 2011;21:1102–10. <https://doi.org/10.1007/s00330-010-1998-x>.

S2. Manganaro L, Lakhman Y, Bharwani N et al. Staging, recurrence and follow-up of uterine cervical cancer using MRI: Updated Guidelines of the European Society of Urogenital Radiology after revised FIGO staging 2018. Eur Radiol 2021;31:7802–16. https://doi.org/10.1007/s00330-020-07632-9.


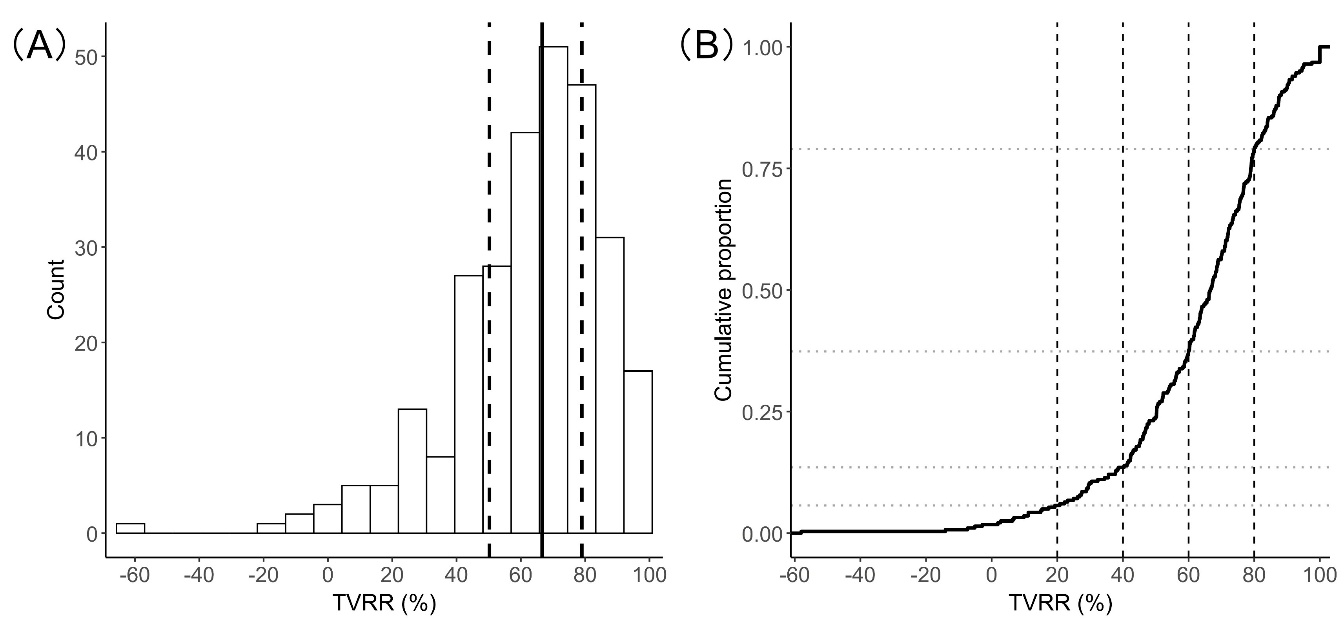


Supplementary Figure S1. Distribution of TVRR. (A) Histogram of TVRR. The solid vertical line shows the cohort median and the dashed lines are the IQR (25th and 75th percentiles). (B) Empirical cumulative distribution of TVRR. Vertical dashed lines indicate 20%, 40%, 60%, and 80%. The y-axis shows the cumulative proportion. In the cohort (N=281), the median TVRR was 66.6% (IQR 50.3–79.0%); and the proportions with TVRR ≥20%, ≥40%, ≥60%, and ≥80% were 94.3%, 86.5%, 62.6%, and 21.0%, respectively.


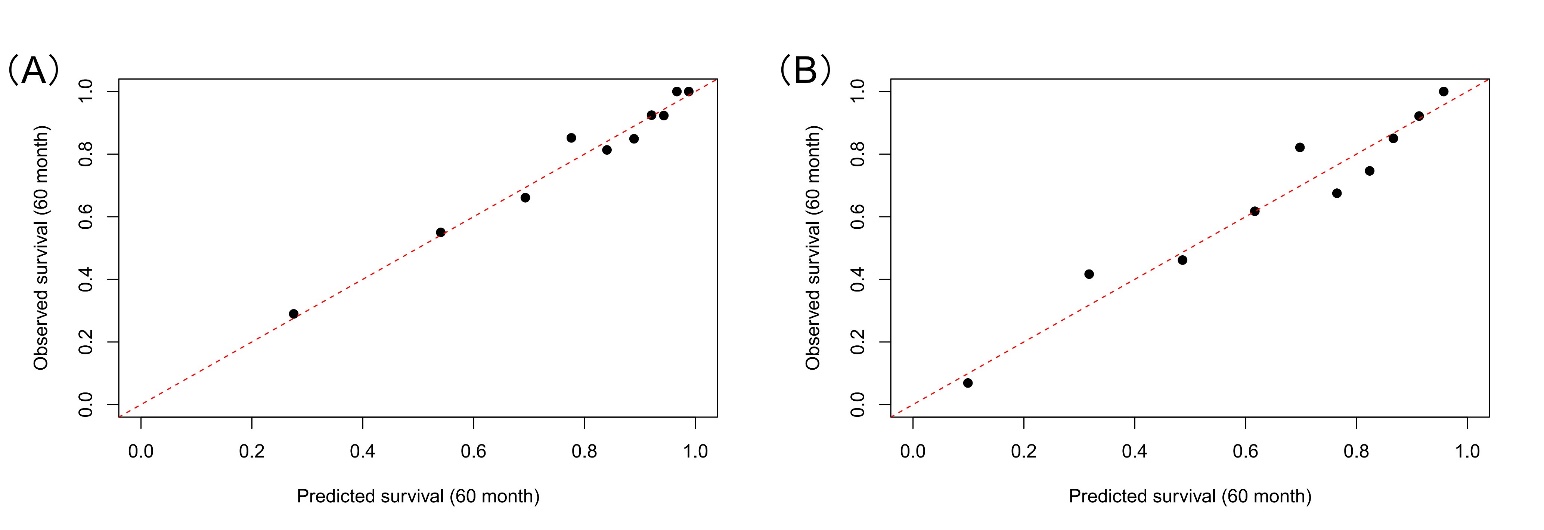


Supplementary Figure S2. Calibration of the Cox models at 60 months. Decile-grouped calibration for OS (A) and PFS (B). Predicted survival at 60 months was obtained from the Cox baseline survival evaluated at 60 months combined with each patient’s linear predictor; observed survival was estimated by Kaplan-Meier within deciles of predicted risk. Points show decile means (predicted vs. observed). The dashed line indicates ideal calibration (y=x).


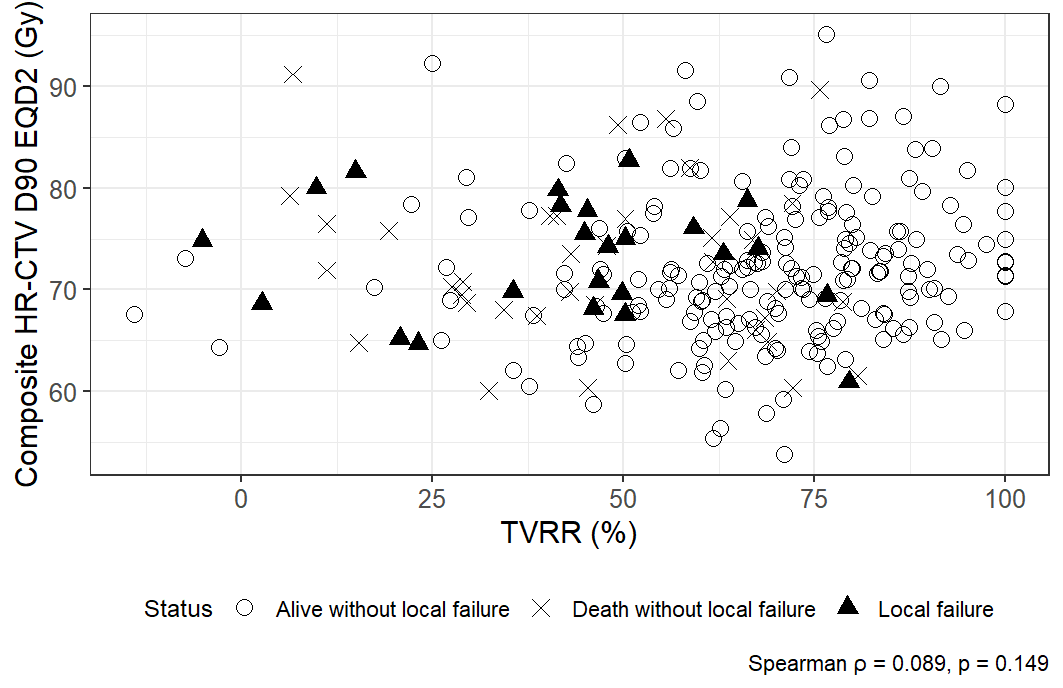


Supplementary Figure S3. Scatter plot of TVRR versus composite HR-CTV D90 EQD2. Each point represents an individual patient. Open circles indicate patients alive without local failure; crosses indicate patients who died without local failure (competing event); filled triangles indicate patients with local failure. Fifteen patients treated with 2D planning were excluded due to unavailable HR-CTV D90 values (n = 266). Spearman's rank correlation coefficient was 0.089 (p = 0.149), indicating no significant correlation between TVRR and composite HR-CTV D90 EQD2.

Supplementary Table S1. Generalizability of study population(s): Blinded version

| Condition | Description | |
| --- | --- | --- |
| Disease, problem, or condition under investigation | Definitive chemoradiation for FIGO 2009 stage IB-IVA cervical cancer, with mid-treatment pelvic MRI during EBRT (window 24-36 Gy) to derive the tumor volume reduction rate (TVRR). | |
| Relevant considerations of disease, problem, or condition in relation to: | *Note any relevant considerations in boxes below:* | |
| Sex and gender | Female only; gender identity not collected | |
| Age | Median age 59 years (IQR 49–66) | |
| Race or ethnic group | Not formally collected; population drawn from a relatively homogeneous regional population; specific details withheld for peer review. | |
| Geography |  | |
| Other considerations | Volumetry required adequate T2-weighted MRI in two planes. TVRR calculated from blinded, protocolized contours with consensus review as needed. | |
| Study | Description | |
| Overall assessment of generalizability of the study population | | - The cohort reflects typical locally advanced cervical cancer treated with contemporary regional practice (3D-CRT with central shielding, CT-based IGABT with interstitial capability, weekly cisplatin) at two regional centers; transportability is strongest to similar RT/BT programs. - Requirements for image quality and protocolized volumetry may limit applicability if mid-treatment MRI or contouring expertise is not available. - Internal validation and cross-center IECV metrics are summarized in Results 3.5 and Table 2. |

Abbreviations: FIGO, International Federation of Gynecology and Obstetrics; MRI, magnetic resonance imaging; EBRT, external beam radiotherapy; TVRR, tumor volume reduction rate; IQR, interquartile range; 3D-CRT, 3-dimensional conformal radiation therapy; CT, computed tomography; IGABT, image-guided adaptive brachytherapy; IECV, internal-external cross‑validation.

Supplementary Table S2. Model specifications used across analyses. Cross-walk indicating which specification was used for each endpoint and analysis.

| Purpose / used in | Endpoint | Specification name | TVRR term | Covariates |
| --- | --- | --- | --- | --- |
| Primary analysis;  internal validation | OS | Full specification (OS) | Spline  (nonlinearity tested on 3 df) | Forced: age, FIGO 2009 stage, nodal status, histology; AIC-selected: pre-treatment Hb |
| Primary analysis; internal validation | PFS | Full specification  (PFS) | Spline  (nonlinearity tested on 3 df) | Forced: age, FIGO 2009 stage, nodal status, histology; AIC-selected: pre-treatment Hb, OTT |
| Primary analysis;  internal validation | LC | Full specification (Fine–Gray, LC) | Spline  (nonlinearity tested on 3 df) | Forced: FIGO 2009 stage, histology, pre‑treatment tumor volume |
| IECV (train one center, test the other) | OS / PFS | Core specification | Linear | Forced: age, FIGO 2009 stage, nodal status, histology |
| Effect modification analyses (by center) | OS/ PFS | Core specification + TVRR × center interaction | Linear | Forced: age, FIGO 2009 stage, nodal status, histology |
| Sensitivity analysis; web calculator | OS | Implementation (linear) specification | Linear | Same as full specification (OS) |
| Sensitivity analysis; web calculator | PFS | Implementation  (linear) specification | Linear | Same as full specification (PFS) |

Units/scaling: TVRR per 10 pp; age per 10 years; pre-treatment Hb per 1 g/dL; OTT per 7 days; pre‑treatment tumor volume per 10 cm³.

Abbreviations: TVRR, tumor volume reduction rate; OS, overall survival; PFS, progression‑free survival; LC, local control; df, degree of freedom; FIGO, International Federation of Gynecology and Obstetrics; Hb, hemoglobin; OTT, overall treatment time; IECV, internal-external cross‑validation; AIC, Akaike’s Information Criterion.

Supplementary Table S3. Linearized multivariable models and baseline survival landmarks.

1. Multivariable Cox model (OS): linear TVRR specification

| Variables (definition) | beta | SE | z | p-value | HR (95% CI) |
| --- | --- | --- | --- | --- | --- |
| TVRR - linear term (per 10-pp increase) | -0.342 | 0.045 | -7.66 | <0.001 | 0.71 (0.65–0.78) |
| Age (per 10 years increase) | 0.183 | 0.118 | 1.55 | 0.121 | 1.20 (0.95–1.52) |
| Histology (non-SCC vs. SCC (ref)) | 0.428 | 0.305 | 1.40 | 0.161 | 1.53 (0.84–2.79) |
| FIGO 2009 stage (Stage III-IV vs. I-II (ref)) | 0.678 | 0.330 | 2.06 | 0.040 | 1.97 (1.03–3.76) |
| Nodal status (positive vs. negative (ref)) | 0.618 | 0.286 | 2.16 | 0.031 | 1.86 (1.06–3.25) |
| Pre-treatment hemoglobin (per 1 g/dL increase) | -0.149 | 0.077 | -1.92 | 0.055 | 0.86 (0.74–1.00) |

(B) Multivariable Cox model (PFS): linear TVRR specification

| Variables (definition) | beta | SE | z | p-value | HR (95% CI) |
| --- | --- | --- | --- | --- | --- |
| TVRR - linear term (per 10-pp increase) | -0.295 | 0.038 | -7.82 | <0.001 | 0.74 (0.69–0.80) |
| Age (per 10 years increase) | 0.050 | 0.092 | 0.54 | 0.586 | 1.05 (0.88–1.26) |
| Histology (non-SCC vs SCC (ref)) | 0.738 | 0.240 | 3.07 | 0.002 | 2.09 (1.31–3.35) |
| FIGO 2009 stage (Stage III-IV vs. I-II (ref)) | 0.402 | 0.244 | 1.65 | 0.100 | 1.50 (0.93–2.41) |
| Nodal status (positive vs. negative (ref)) | 0.811 | 0.234 | 3.46 | <0.001 | 2.25 (1.42–3.56) |
| Pre-treatment hemoglobin (per 1 g/dL increase) | -0.101 | 0.063 | -1.60 | 0.109 | 0.90 (0.80–1.02) |
| Overall treatment time (per 7-day increase) | 0.241 | 0.116 | 2.08 | 0.038 | 1.27 (1.01–1.60) |

(C) Baseline survival and cumulative baseline hazard at prespecified landmarks.

| Time (months) | S_0_, OS | H_0_, OS | S_0_, PFS | H_0_, PFS |
| --- | --- | --- | --- | --- |
| 12 | 0.908 | 0.0970 | 0.839 | 0.1761 |
| 36 | 0.492 | 0.7091 | 0.712 | 0.3393 |
| 60 | 0.309 | 1.1757 | 0.633 | 0.4566 |

Application of coefficients (Cox): For a subject with linear predictor η = xβ, the cumulative hazard at time t is H(t|x) = H₀(t)·exp(η), and survival is S(t|x) = [S₀(t)]^{exp(η)}.

(D) Predicted 5-year absolute risks for representative TVRR values

| **TVRR (%)** | **5-year OS (%)** | **5-year PFS (%)** |
| --- | --- | --- |
| 30 | 45.8 | 28.3 |
| 40 | 57.4 | 39.1 |
| 50 | 67.4 | 49.7 |
| 60 | 75.6 | 59.4 |
| 70 | 82.0 | 67.9 |
| 80 | 86.8 | 74.9 |

Reference covariate profile: age 59 years, SCC histology, FIGO 2009 stage III–IV, node-positive, pre-treatment hemoglobin 11.8 g/dL, overall treatment time 46 days (cohort median/modal values). Predicted probabilities were computed from the linearized Cox models (panels A and B) and baseline survival at 60 months (panel C). For other covariate combinations, see the Supplementary Web Calculator.

Abbreviations: TVRR, tumor volume reduction rate; SE, standard error; HR, hazard ratio; CI, confidence interval; pp, percentage points; ref, reference; SCC, squamous cell carcinoma; FIGO, International Federation of Gynecology and Obstetrics; OS, overall survival; PFS, progression-free survival.

Supplementary Table S4. Sensitivity analyses

| (A) | Overall survival  (Events 38/171) | | Progression-free survival  (Events 56/171) | |
| --- | --- | --- | --- | --- |
|  |  |  |  |  |
| Variables (definition) | HR (95% CI) | Wald χ^2^  (p-value) | HR (95% CI) | Wald χ^2^  (p-value) |
| TVRR - linear term (per 10-pp increase) | 0.73 (0.66–0.81) | 34.5 (<0.001) | 0.77 (0.71–0.84) | 33.5 (<0.001) |
| Age (per 10 years increase) | 1.15 (0.86–1.54) | 0.9 (0.339) | 1.02 (0.81–1.30) | 0.0 (0.848) |
| Histology (non-SCC vs. SCC (ref)) | 1.92 (0.79–4.66) | 2.1 (0.151) | 3.03 (1.52–6.05) | 9.9 (0.002) |
| FIGO 2009 (Stage III-IV vs. I-II (ref)) | 1.88 (0.85–4.15) | 2.4 (0.119) | 1.20 (0.66–2.19) | 0.4 (0.554) |
| Nodal status (positive vs. negative (ref)) | 2.38 (1.11–5.11) | 5.0 (0.026) | 2.27 (1.24–4.15) | 7.1 (0.008) |
| Pre-treatment hemoglobin (per 1 g/dL increase) | 0.82 (0.68–1.00) | 3.7 (0.053) | 0.89 (0.76–1.04) | 2.1 (0.149) |
| Overall treatment time (per 7-day increase) | — | — | 1.17 (0.84–1.63) | 0.9 (0.342) |
| Discrimination (C-index) | 0.77 | | 0.76 | |

| (B) | Overall Survival  (Events 39/214) | | Progression-free Survival  (Events 57/214) | |
| --- | --- | --- | --- | --- |
|  |  |  |  |  |
| Variables (definition) | HR (95% CI) | Wald χ^2^  (p-value) | HR (95% CI) | Wald χ^2^  (p-value) |
| TVRR - linear term (per 10-pp increase) | 0.70 (0.62–0.80) | 27.9 (<0.001) | 0.74 (0.66–0.83) | 27.9 (<0.001) |
| Age (per 10 years increase) | 1.29 (0.96–1.73) | 2.9 (0.089) | 1.09 (0.86–1.38) | 0.5 (0.491) |
| FIGO 2009 (Stage III-IV vs. I-II (ref)) | 1.84 (0.85–4.01) | 2.4 (0.122) | 1.20 (0.67–2.17) | 0.4 (0.535) |
| Nodal status (positive vs. negative (ref)) | 1.61 (0.79–3.25) | 1.7 (0.188) | 2.42 (1.33–4.41) | 8.4 (0.004) |
| Pre-treatment hemoglobin (per 1 g/dL increase) | 0.78 (0.64–0.94) | 6.4 (0.011) | 0.90 (0.77–1.05) | 1.9 (0.167) |
| Overall treatment time (per 7-day increase) | — | — | 1.34 (0.95–1.89) | 2.9 (0.091) |
| Discrimination (C-index) | 0.74 | | 0.72 | |

| (C) Subset | Endpoint | TVRR HR (subset;  per 10 pp) | 95% CI (subset) | TVRR HR (remainder; per 10 pp) | 95% CI (remainder) | Interaction p (TVRR×subset) |
| --- | --- | --- | --- | --- | --- | --- |
| MRI window | OS | 0.72 | 0.65–0.80 | 0.69 | 0.59–0.82 | 0.722 |
| MRI window | PFS | 0.75 | 0.69–0.82 | 0.73 | 0.64–0.83 | 0.672 |
| SCC + cisplatin + IGABT | OS | 0.70 | 0.62–0.80 | 0.72 | 0.63–0.81 | 0.851 |
| SCC + cisplatin + IGABT | PFS | 0.73 | 0.65–0.82 | 0.75 | 0.68–0.84 | 0.688 |

| (D) | Overall Survival  (Events 59/266) | | Progression-free Survival  (Events 90/266) | |
| --- | --- | --- | --- | --- |
|  |  |  |  |  |
| Variables (definition) | HR (95% CI) | Wald χ^2^  (p-value) | HR (95% CI) | Wald χ^2^  (p-value) |
| TVRR - linear term (per 10-pp increase) | 0.70 (0.63–0.78) | 43.0 (<0.001) | 0.74 (0.68–0.80) | 47.4 (<0.001) |
| Age (per 10 years increase) | 1.23 (0.96–1.58) | 2.8 (0.097) | 1.04 (0.86–1.25) | 0.2 (0.697) |
| FIGO 2009 (Stage III-IV vs. I-II (ref)) | 2.24 (1.10–4.58) | 4.9 (0.027) | 1.55 (0.93–2.57) | 2.9 (0.091) |
| Nodal status (positive vs. negative (ref)) | 1.99 (1.11–3.59) | 5.3 (0.022) | 2.38 (1.47–3.84) | 12.5 (<0.001) |
| Pre-treatment hemoglobin (per 1 g/dL increase) | 0.86 (0.74–1.01) | 3.4 (0.066) | 0.91 (0.80–1.03) | 2.3 (0.129) |
| Overall treatment time (per 7-day increase) | — | — | 1.28 (1.00–1.64) | 3.8 (0.053) |
| HR-CTV D90 EQD2 (per 10-Gy increase) | 1.24 (0.88–1.75) | 1.5 (0.216) | 1.16 (0.87–1.55) | 1.1 (0.305) |
| Discrimination (C-index) | 0.80 | | 0.76 | |

Panels (A) and (B) correspond to two subsets: (A) combined imaging-timing subset (pre-treatment MRI ≤28 days before RT start and mid-treatment MRI at 26–30 Gy); and (B) SCC treated with weekly concurrent cisplatin and CT-based IGABT.

Panel C summarizes heterogeneity using an interaction model fitted in the full analysis population (TVRR×subset) with the same covariates. It reports the TVRR effect in each subset and its remainder, and the interaction p-value (likelihood-ratio test).

Panel (D) adds HR-CTV D90 EQD2 (composite EBRT + BT dose) as a covariate, using the implementation (linear) specification. Fifteen patients (5%) lacking HR-CTV D90 due to 2D planning were excluded.

Abbreviations: TVRR, tumor volume reduction rate; HR, hazard ratio; CI, confidence interval; pp, percentage points; SCC, squamous cell carcinoma; FIGO, International Federation of Gynecology and Obstetrics; IGABT, image-guided adaptive brachytherapy; HR-CTV D90, Minimum doses to 90% of the high-risk clinical target volume; EQD2, equivalent dose in 2-Gy fractions; EBRT, external‑beam radiotherapy; BT, brachytherapy.
