# Supplementary material for: Mid-treatment MRI-based tumor volume reduction rate as a continuous prognostic factor after chemoradiation for cervical cancer: development and two-center internal–external validation
Source: J Radiat Res. 2026 Jun 25;67(4):634–47. doi: 10.1093/jrr/rrag043 (PMC13400566; doi:10.1093/jrr/rrag043)
Supplement: Supplementary_material_rrag043 [file supplementary_material_rrag043.zip › Supplementary Web Calculator.zip!/HTML_calc.html]

TVRR-based Survival Calculator (OS / PFS)


# TVRR-based Survival Calculator (OS / PFS) — Implementation (linear)

#### Clinical inputs

TVRR (%)

Age (years)

Histology

SCC (ref)
non-SCC

FIGO 2009 stage

I–II (ref)
III–IV

Nodal status

Negative (ref)
Positive

Pre-treatment hemoglobin (g/dL)

Overall treatment time (days) [used in PFS only]

Units: TVRR per 10-pp, Age per 10 years, Hb per 1 g/dL, OTT per 7 days. OS has no OTT term.

#### Linear predictors (η)

Formula: S(t|x) = [S0(t)]^{exp(η)}. Reference levels: SCC, FIGO I–II, node negative.

#### Overall Survival (OS)

| Time (mo) | Survival | Event risk |
| --- | --- | --- |

#### Progression-free Survival (PFS)

| Time (mo) | Survival | Event risk |
| --- | --- | --- |


Download CSV (inputs + predictions)

#### Model details (coefficients, metadata)

#### Coefficients in use (Implementation, linear)

| Coefficient | OS | PFS |
| --- | --- | --- |

**Spec:** Implementation (linear) (OS: +Hb; PFS: +Hb + OTT)   |  
**Version:**    |  
**Date:**    |  
**DOI:**

Research/educational use only. Not a medical device. **No inputs are stored.**
Do not enter personally identifiable information (PII). External validation pending.
